# Molecular Signature of HPV-Induced Carcinogenesis: pRb, p53 and Gene Expression Profiling

**DOI:** 10.2174/138920209787581235

**Published:** 2009-03

**Authors:** Águeda Buitrago-Pérez, Guillermo Garaulet, Ana Vázquez-Carballo, Jesús M Paramio, Ramón García-Escudero

**Affiliations:** Molecular Oncology Unit, Molecular Biomedicine Division, CIEMAT, Ave. Complutense 22, E-28040 Madrid, Spain

**Keywords:** Human papillomavirus, E6, E7, cervical cancer, oropharyngeal cancer, gene expression profiling, pRb, p53.

## Abstract

The infection by mucosal human papillomavirus (HPV) is causally associated with tumor development in cervix and oropharynx. The mechanisms responsible for this oncogenic potential are mainly due to the product activities of two early viral oncogenes: E6 and E7. Although a large number of cellular targets have been described for both oncoproteins, the interaction with tumor suppressors p53 and retinoblastoma protein (pRb) emerged as the key functional activities. E6 degrades tumor suppressor p53, thus inhibiting p53-dependent functions, whereas E7 binds and degrades pRb, allowing the transcription of E2F-dependent genes. Since these two tumor suppressors exert their actions through transcriptional modulation, functional genomics has provided a large body of data that reflects the altered gene expression of HPVinfected cells or tissues. Here we will review the similarities and differences of these findings, and we also compare them with those obtained with transgenic mouse models bearing the deletion of some of the viral oncogene targets. The comparative analysis supports molecular evidences about the role of oncogenes E6 and E7 in the interference with the mentioned cellular functions, and also suggests that the mentioned transgenic mice can be used as models for HPV-associated diseases such as human cervical, oropharynx, and skin carcinomas.

## INTRODUCTION

Human papillomaviruses (HPV) are the etiological agents responsible for a number of pathologies affecting the stratified epithelia of the skin and anogenital and oropharyngeal sites [1-3]. Specific HPV types (“low risk” HPVs) cause benign warts, but other types (“high risk” HPVs) are strongly associated with premalignant and malignant invasive carcinomas, especially in the uterine cervix. Cervical cancer (CC) is the second most frequent type of women cancer worldwide, being especially important in the developing countries, where gynecological screening is not sufficiently implemented in the clinical routine [3]. Although less frequently, HPV infections have also been associated with carcinoma development in skin and oropharynx. Overall, about 25% of the head and neck squamous cell carcinomas (HNSCCs) are infected with “high risk” HPVs, and display clinical and molecular features that distinguish them from the remaining HNSCCs [4].

Multiple molecular studies have reported that HPV-mediated carcinogenesis is mainly due to the oncogenic activities of the viral early proteins E6 and E7. E6 is able to induce the degradation of p53 *via *direct binding to the ubiquitin ligase E6AP, inhibiting p53-dependent signaling upon stress stimuli, and contributing to tumorigenesis [5-7]. On the other hand, E7 oncoprotein E7 associates with the retinoblastoma family of proteins (pRb, p107 and p130) and disrupts their association with the E2F family of transcription factors, subsequently transactivating cellular proteins required for cellular and viral DNA replication. Moreover, E7 binds and induces the proteasomal-mediated degradation of all pRb, p107 and p130 proteins [8-10]. p53 and pRb are well-known cellular tumor suppressors involved in processes such as cell cycle progression, DNA repair, apoptosis, differentiation, senescence, and chromatin remodeling. Very importantly, in the vast majority of human tumors pRb and/or p53 functions are impaired.

Recently, preventive vaccination against HPV infections has been launched in some countries, protecting from most of the benign and malignant lesions in the cervix. Epidemiological studies estimate that about 10% of the women population is already infected with “high risk” mucosal HPVs in the genital area, population in which vaccination would not be worthy, as it is not curative. Although the infection is not sufficient (but necessary) for tumor development, it is important to develop curative therapies for HPV-related carcinogenesis [11].

Gene expression profiling analysis using DNA microarray technologies has emerged in the last decade as a powerful tool to search for reliable gene markers of specific cancers, to determine the molecular features than distinguished or grouped different histological types of cancers, and to correlate specific gene expression patterns with clinical outcome [12]. Moreover, microarray analysis provides a powerful tool to compare molecular features of mouse models of human cancer and their human counterparts [12]. A large number of reports have described gene expression deregulation of cervical cancer and HPV-infected or non-infected HNSCC [13-23]. Interestingly, some of the deregulated genes observed in the infected tumors have been described in previous studies of *in vitro* HPV-models, either from basic analysis of viral oncogenic mechanisms or from gene expression profiling analysis [24-31].

In this review, we compare data of deregulated genes from HPV-infected human cervical and oropharyngeal tumors, *in vitro* cell models of oncogenic HPV, and animal mouse models bearing the deletion of retinoblastoma family and/or p53 genes. The comparison helps to define molecular similarities between cervical and oropharyngeal HPV-infected tumors, and also between the human tumors and different models of the HPV-associated disease. The report also emphasizes the need to perform simultaneous analysis of multitude of HPV-infected and non-infected cervical and oropharyngeal carcinomas, with full clinical information in order to extract accurate molecular signatures associated with HPV-infection, and/or with clinical outcome.

## PATTERNS OF OVEREXPRESSED GENES IN HPV-INFECTED TUMORS

Gene expression profiles of HPV-infected human tumors with normal, uninfected tissue have been described. Most of these studies have been done on CC, although some have been performed in HNSCC. These analyses have allowed authors to find genes related to HPV-related tumor progression or expression patterns of HPV-related tumors. As expected, some of the genes described in these comparisons are well known markers of infection as previously described in basic molecular studies of papillomavirus oncogene activities, or in screening of markers using human clinical samples. Generally, gene expression deregulation caused by papillomavirus infection is related to cell cycle genes, most of them E2F genes and E2F-regulated genes. These findings are in agreement with oncogenic activities of the E7 viral early gene, able to bind and inactivate members of the retinoblastoma proteins pRb, p107 and p130, which act as repressors of E2F transcription factors. However, expression profiling has helped to delimitate other previously unknown genetic factors related to HPV-induced carcinogenesis, some of which could represent markers of virus-mediated malignancy and/or targets for therapeutic intervention.

In order to find common hallmarks of HPV related carcinogenesis, we decided to compare published gene lists of microarray analyses. Firstly, we focused on the studies performed in cervical cancer, whereby normal cervical tissue or low-grade squamous intraepithelial lesion (LSIL) were compared with high grade squamous intraepithelial lesions (HSIL) or with CC. Up to seven reports were used for comparison (Table **1**), performed in three different microarray platforms. We selected those genes that were overexpressed in at least 3 out of 7 reports, giving rise to 14 common genes of HPV-related cervical cancer (Signature 1, see Table **2**). If we extend the analysis including a report whereby normal oral tissue was compared to HPV-infected oropharyngeal carcinomas, new common genes appear in at least 3 of the studies, resulting in a group of 28 genes (Signature 2). This signature represents genes differentially expressed between normal, non-malignant samples and HPV-infected carcinomas from mucosal tissues.

In a second analysis, we performed a comparison between HPV-infected *versus* non infected HNSCC from 3 different studies using two distinct platforms. Only 7 genes displayed similar results in at least 2 studies (Signature 3, Table **2**), representing genes that could distinguish HPV-related induced carcinogenesis from non viral mechanisms of HNSCC tumorigenesis. If we include in the comparison an analysis of the genes deregulated in HPV-infected carcinomas of both CC and HNSCC, the number of common genes in at least 2 studies increased to 35 (Signature 4, Table **2**). Of note, none of the genes obtained in this comparison is present in the Signature 1, suggesting that major differences between HPV-positive and negative cancers are distinguishable from the differences between normal and tumoral tissue. However, shared molecular determinants of both comparisons can be limited, since we compare the reported expression lists of deregulated genes. However, less than half of the described reports have allocated raw data of the complete gene expression data in public repositories, thus precluding the collection of complete records of the published studies and the differential expression analysis in a multiexperiment dataset.

In a third analysis, we decided to perform a global comparison of molecular similarities between normal *versus* HPV-tumoral samples and HPV-infected *versus* uninfected samples of all mentioned reports. Signature 5 (Table **2**) contains 26 genes overexpressed in 3 or more comparisons out of 12, representing a HPV-infected mucosal carcinoma signature. Detailed functional analysis of the HPV-tumor infected signature (Signature 5) using enrichment of Gene Ontology biological processes associated with the 26 genes evidenced that virus induces a clear deregulation in cell cycle genes, as it has been already described. Fig. (**1**) shows the result of the functional analysis performed using DAVID Functional Annotation web tool [32, 33], where the most relevant enriched terms are included. Genes involved in DNA replication, DNA repair, cell cycle control or mitosis are present. Interestingly, they also appear 3 genes involved in proteolysis within the term “ubiquitin-dependent protein catabolic process”: USP1, UBE2C and PSMB9.

## PATTERNS OF UNDEREXPRESSED GENES IN HPV-INFECTED TUMORS

From the studies that have been used to check for common overexpressed genes, there are only 7 comparisons containing repressed genes, being 4 comparisons of normal *versus* carcinoma samples (3 CC and 1 HNSCC) and 3 comparisons of HPV-positive *versus* negative samples. Only an analysis of common genes between all of them was possible, resulting in a number of 31 genes downregulated in 2 or more comparisons out of 7 (Signature 6). This signature represents genes that are underexpressed in HPV-infected mucosal carcinomas. The functional analysis based on the GO database (Fig. (**1**)) showed that the number of enriched functions and their statistical significance is lower than the observed for up-regulated genes. Proteases belonging to the family of kallikreins (KLK7, KLK8, KLK10, KLK11 and KLK13) are highly represented in a term named “proteolysis”. Other interesting functions are some related to “epidermis development” or “keratinocyte differentiation”.

## HPV-INFECTED MUCOSAL CARCINOMA SIGNATURE DURING CERVICAL CARCINOMA PROGRESSION

Gius and collaborators [26] have reported an analysis of the changes in genomic expression that occur as normal cervical tissue progresses to carcinoma *in situ* (CIN) an then to cervical carcinoma, in either epithelium or stromal compartments using laser capture microdissection. Up to 5 genes belonging to the Signature 5 were reported to be overexpressed during cancer progression, but exclusively within the epidermal compartment: CDKN2A, CENPF, MCM2, RFC4 and TK1. On the other hand, downregulated genes in epidermal cells during malignant progression that are present in the HPV-tumor associated Signature 6 were: KLK11, CDA, CRABP2, CDA and KIF1C. These results corroborates the HPV-tumor associated deregulation of some of the genes of the Signatures 5 and 6 (Table **3**), and demonstrate that these genes are particularly deregulated into the epidermal (but no stromal) component of the carcinomas.

## *IN VITRO* SYSTEMS FOR HPV-INDUCED GENE EXPRESSION DEREGULATION

A few reports have described the effect of viral oncogene expression in cellular or organotypic cultures. Duffy and collaborators used human foreskin primary keratinocytes retrovirally transduced with both HPV16 E6 and E7 oncogenes, or E7 in the presence of 2 different E6 mutants [24]. Comparison of genes described as differentially expressed in the transduced cells showed no overlap with Signature 5 of HPV-induced carcinomas. On the other hand, comparison of underexpressed genes showed an overlap of 4 genes with Signature 6 (KLK7, SULT2B1, ECM1 and LY6D) (Table **3**). Furthermore, Wan and collaborators performed a similar analysis, but using immortalized human primary keratinocytes transfected with the head-to-tail dimer of HPV16 DNA [31]. Comparisons of genes deregulated with normal primary cells gave rise to an overlap of only 1 overexpressed gene with Signature 5 (CDKN2A) and 6 underexpressed genes with Signature 6 (KLK10, KLK11, SCEL, KLK7, LY6D and S100P) (Table **3**). Results suggest that the cellular systems used could not mimic gene overexpression of HPV-associated carcinomas, although they could induce some of the expression patterns of downregulated genes.

Furthermore, Garner-Hamrick and collaborators reported a microarray analysis of organotypic cultures of human primary keratinocytes retrovirally transduced with the E6 and E7 oncogenes of HPV18 under the control of the native differentiation-dependent HPV enhancer-promoter [25]. Results showed overlaps of over- and under-expressed genes with the comparative Signatures 5 and 6, respectively (Table **3**), suggesting that this analysis model is more similar to HPV-related carcinomas, possibly because the proliferation/differentiation proccesses of the epidermal cells are better resembled than in cellular non-organotypic cultures.

Finally, a number of reports have described that the inhibition of expression of HPV18 E6/E7 genes inserted into the HeLa cell genome induced important gene deregulation leading to growth inhibition and/or cellular senescence [34-38]. Kuner and collaborators described gene expression changes after RNA interference of E6 and E7 oncogenes, showing that gene viral interference in HeLa cells repressed genes normally induced in cervical tumors, and induced genes that appear downregulated in tumors [27]. Comparison with our HPV-signatures revealed similar findings. Thus, Signature 5 genes as CENPF, DTL, EZH2, KIF15, MCM2, MELK and PSMB9 are repressed after RNA interference; conversely, only HIST2H3PS2, that is normally repressed in HPV-tumors, was also induced after viral oncogene inhibition (Table **3**). The comparison corroborates that E6 and E7 oncogene viral activity in HeLa cancer cell line is essential to maintain molecular determinants of HPV-associated tumorigenesis.

## MOUSE MODELS OF SKIN CARCINOMAS AND SIMILARITIES WITH HPV CARCINOGENESIS

Recently, using mouse models we have described the phenotypical consequences of the deletion of members of the retinoblastoma protein family in the skin homeostasis. Mice with conditional deletion of Rb alleles in stratified epithelia (K14Cre; Rb^loxP/loxP^, thereafter pRb- mice) display increased and aberrant cell proliferation, impaired differentiation, and the disengagement of these processes as cells committed in the differentiation process are still able to proliferate *in vivo* and *in vitro* [39]. These differences in phenotype are more severe with the loss of p107 in K14Cre; Rb^loxP/loxP^; Rbl1-/- (pRb-; p107-) mice [39]. However, although p107 and p130 exert overlapping functions in epidermis [40], K14Cre; Rb^loxP/loxP^; Rbl2-/- (pRb-; p130-) mice were very similar to Rb- mice [28]. Importantly, we could correlate the proliferation differences with specific changes in gene overexpression between pRb-, pRb-; p107- and pRb-; p130- primary keratinocytes using microarray analysis (pRb/p107/p130 signature), and explain the phenotypes in the context of altered E2F expression and functionality [28]. Comparison of the mouse skin pRb/p107/p130 signature mapped to human genes with Signature 5 of mucosal HPV-carcinomas revealed a significant overlap of 7 genes: DHFR, E2F1, LIG1, MCM2, PCNA, RFC4, TYMS and USP1.

On the other hand, although pRb-; p107- mice die soon after birth, we have been able to demonstrate that skin transplants from pRb-; p107- onto immunosupressed mice induce spontaneous tumors [29]. Again, using microarray analysis of pretumoral skin, we have shown that double deletion of pRb and p107 produces the reduction of p53-dependent pro-apoptotic signals, and the increased of E2F-dependent genes [29, 41]. Overall, our data demonstrated that p107 behaves as a tumor suppressor in epidermis in the absence of pRb. We can argue that the overlap observed between pRb/p107/p130 mouse skin signature and the human Signature 5 could be partially due to combined loss of pRb, p107, and functional inhibition of p53. We proposed, thus, that pRb-; p107- mouse model could be used as a tool to analyze oncogenic activities of “high risk” HPVs.

On the other hand, we have demonstrated that K14Cre; Rb^loxP/loxP^; TP53^loxP/loxP^ mice (pRb-; p53-) and K14Cre; TP53^loxP/loxP^ mice (p53-) develop highly undifferentiated and very aggressive spontaneous squamous cell carcinoma in the skin [30] that display a high lung metastatic capacity (submitted). A gene expression microarray comparison between normal skin and primary tumors from both genotypes have allowed us to extract p53-deficient tumor signatures of overexpressed and underexpressed genes (unpublished data). Human mapped genes revealed an overlap of 7 overexpressed genes with those present in HPV-tumors of Signature 5: CDC7, ECT2, MCM2, TK1, TPX2, TYMS and UBE2C. Importantly, these genes were commonly overexpressed in pRb-; p53- as well as p53- tumors, highlighting that deletion of only the TP53 alleles resulted in tumors displaying similar deregulation than those arising by combined Rb and TP53 deletion. Again, these mouse models do exhibit similar molecular trends than HPV-human tumors, and could possibly be preclinical tools for therapeutic intervention of aggressive forms of the disease.

## GENE OVEREXPRESSION IN HPV-TUMORS

Many reports have described that HPV-associated tumor samples are characterized by the overexpression of cell cycle genes. These results are in line with our comparative analysis, in which from a list of 26 genes (Signature 5) commonly overexpressed in HPV-carcinomas, half of them are directly implicated in different processes of the cell cycle (CDKN2A, MYBL2, LIG1, TPX2, CENPF, E2F1, SYCP2, PCNA, MCM2, CDC7, CDKN2C, KIF15, and UBE2C), such as DNA replication, DNA repair, and cell cycle regulation (Fig. (**1**)). Moreover, there are overexpressed genes involved in all phases of cell cycle (G1, G1 to S, and M), which suggests that a general deregulation of cellular proliferation is taking place in infected tissues. Strikingly, genes that have been described to be regulated by active E2F1, as well as E2F1 itself, are upregulated (MYBL2, MCM2, CDC7, DHFR, PCNA, TYMS, EZH2, CDKN2A, and CDKN2C) [42-49]. Moreover, there are also genes that contain E2F-responsive elements within their promoter sequences but for which no transcriptional regulation has yet been described (DTL, KIF15, LIG1, MELK, and SYCP2). These findings demonstrate that pRb-functional interference activity of E7 is actively taking place in tumors, as the gene deregulation elicited in the HPV-carcinomas is mainly due to E2F activity. On the other hand, there are a number of overexpressed genes that are also p53-responsive, suggestive of an interference with p53 pathway function: MYBL2, EZH2, TYMS, DHFR, and ECT2 [50-54]. Interestingly, all but ECT2 are genes repressed by p53 and pRb, which suggests that functional inactivation of both tumor suppressors is taking place.

## UNDEREXPRESSED GENES IN HPV-TUMORS

Within the Signature 6 genes, it is important to remark the presence of various proteases, such as carboxypeptidase CPA4, but mostly serine proteases (PRSS3 and kalikreins), as well as a serine protease inhibitor SERPINB4. Between the serine proteases, 5 members of the kalikrein protein family are included: KLK7, KLK8, KLK10, KLK11 and KLK13. Some of these kalikrein proteins have been described as markers of carcinomas from different organs, although the function for all the members is not yet known. However, it has been described that KLK7 is involved in the degradation of adhesive proteins of the extracellular part of the corneodesmosomes, the junctional structures that mediate corneocyte cohesion, such as corneodesmosin, desmoglein 1, and desmocollin 1 [55]. As the degradation of these proteins at the epidermis surface is necessary for desquamation, the observed deregulation suggests that HPV could modulate the degradation of these proteins in order to get a proper assembly/release of virus particles during desquamation. On the other hand, two proteins whose expression activates the p53-pathway are also underexpressed: PITX2 and EIF5A [56, 57]. PITX2 encoded protein acts as a transcription factor of the RIEG/PITX homeobox family and is involved in Wnt, FGF and TGF-β signaling [58-61]. Importantly, Pitx2a isoform can bind to HPV E6 protein and inhibit E6/E6AP-mediated p53 degradation. Thus, the repression of PITX2 contributes to prevent the accumulation of functional p53 protein in HPV infected cells [62]. Eukaryotic translation initiation factor 5A, is induced by p53 [63], and can trigger apoptosis in cancer cells [64]. In conclusion the downregulation of both genes is in line with functional inhibition of p53 mediated by E6 oncoprotein.

## CONCLUSIONS

The present review is a comparative analysis of published list of deregulated genes in relation with high risk HPV-infection or E6/E7 oncogene activities, in order to find common trends between the different studies. We find gene overlaps of over- and under-expressed genes in HPV-infected human mucosal carcinomas that were validated in a model of cervical carcinoma progression [26] and in HeLa cells treated with RNA interference against HPV18 E6 and E7 oncoproteins [27]. The comparative also shows that cellular systems based on primary human keratinocytes expressing HPV-oncogenes do not faithfully mimic gene deregulation observed in human HPV-carcinomas. The organotypic culture is a more similar approach to the *in vivo* setting, probably because it recapitulates proliferation and differentiation processes better than cellular systems. On the other hand, comparison with transgenic mouse models of retinoblastoma family proteins and/or p53 protein ablation from stratified epithelia corroborates that these proteins are functionally inhibited in HPV-samples, as they share similar gene expression trends. Altogether these observations can help to define new targets for the treatment of HPV-mediated carcinogenesis. Furthermore, such possible new treatments can be tested in preclinical trials in the genetically modified mice and in human keratinocyte organotypic cultures as they recapitulate the findings observed in clinical specimens.

## Figures and Tables

**Fig. (1):**
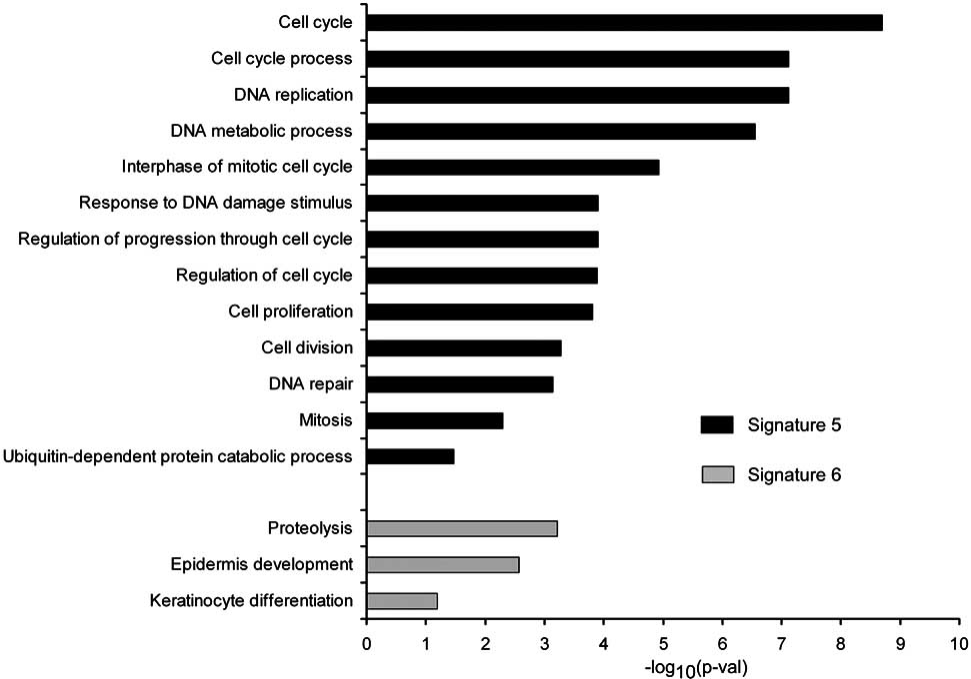
Enrichment analysis of Gene Ontology terms within HPV-infected human mucosal carcinoma signatures. Bar plots represent the statistical significance of the enrichment (-log10(p-val)) in either overexpressed genes (Signature 5, black bars) or underexpressed genes (Signature 6, grey bars).

**Table 1:** Gene Expression Microarray Analysis of Human HPV-Related Cancer

Tissue	Comparative	Number Samples	Gene Cluster	Cluster Size	Reference
Cervix	Normal cervix and LSIL *versus* HSIL, SCC and ACA	17* versus* 17	UP in HSIL, SCC and ACA	46	[13]
Cervix	Normal cervix *versus* cancer cell lines and CC	16* versus* 31	UP in cancer	80	[15]
Cervix	Normal mucosa *versus* cancer cell lines, ACA, and SCC	5* versus* 35	UP in cancer	993	[17]
Cervix	Normal cervix *versus* CC	8* versus* 26	UP in cancer	22	[22]
			DOWN in cancer	5	
Cervix	Normal cervix *versus* CC	18* versus* 29	UP in cancer	21	[21]
			DOWN in cancer	81	
Cervix	Normal cervix and HSIL *versus* CC	17* versus* 21	UP in cancer	29	[23]
			DOWN in cancer	31	
Cervix	Normal cervix *versus* cancer cell lines and CC	20* versus* 29	UP in cancer	29	[19]
Head and neck	Normal oral tissue *versus* oropharyngeal cancer HPV+	4* versus* 3	UP in cancer	220	[14]
			DOWN in cancer	175	
Head and neck	Oropharyngeal HPV+ *versus* oropharyngeal cancer HPV-	3* versus* 4	UP in HPV+	128	[14]
			DOWN in HPV+	40	
Head and neck	HNSCC HPV+ *versus* HNSCC HPV-	12* versus* 30	UP in HPV+	58	[18]
			DOWN in HPV+	61	
Head and neck	HNSCC HPV+ *versus* HNSCC HPV-	8* versus* 28	UP in HPV+	86	[20]
Cervix and Head and neck	CC and HNSCC HPV+ *versus* CC and HNSCC HPV-	43* versus* 18	UP in HPV+	108	[16]
			DOWN in HPV+	10	

**Table 2:** Overlap between HPV-Tumor Microarray Analysis

Signature 1		Signature 2		Signature 3		Signature 4		Signature 5		Signature 6	
Genes	Studies	Genes	Studies	Genes	Studies	Genes	Studies	Genes	Studies	Genes	Studies
APOC1	3	APOC1	3	C7ORF46	2	ANKRD32	2	CDC7	4	KLK10	5
CDKN2A	3	ATP13A3	3	CDKN2C	2	C16ORF75	2	CDKN2A	6	KLK8	4
E2F1	3	BST2	3	CXCR7	2	C5ORF34	2	CDKN2C	4	CRABP2	3
ECT2	3	CDKN2A	4	ITPKB	2	C7ORF46	2	CENPF	3	KLK11	2
MCM2	3	DDIT4	3	MDM1	2	CDC7	2	DHFR	3	KLK13	2
MMP9	3	E2F1	3	SYCP2	3	CDKN2A	2	DTL	4	PRSS3	2
MYBL2	3	ECT2	3	SYNGR3	2	CDKN2C	3	E2F1	3	SCEL	2
PCNA	3	IFI27	3			CENPK	2	ECT2	3	CDA	2
PLOD2	3	MCM2	4			CXCR7	2	EZH2	3	CPA4	2
RFC4	3	MELK	3			DHFR	2	KIF15	3	KLK7	2
SKP2	3	MMP1	3			DTL	2	LIG1	4	SULT2B1	2
TK1	3	MMP12	3			EZH2	2	LY75	4	TGM3	2
TPX2	3	MMP9	3			IL17RB	2	MCM2	5	AREG	2
UBE2C	3	MYBL2	3			ITPKB	2	MELK	3	CD5	2
		NUP62	3			KIF15	2	MYBL2	3	ECM1	2
		PCNA	4			LIG1	2	PCNA	4	EIF5A	2
		PLAT	3			LOC285084	2	PSMB9	4	EPS8L1	2
		PLOD2	3			LOC388494	2	RFC4	5	FGFBP1	2
		PSMB9	3			LOC440331	2	SYCP2	7	GCNT2	2
		RFC4	4			LY75	2	SYNGR3	4	H2BFS	2
		SKP2	3			MDM1	2	TK1	3	HIST2H3PS2	2
		SYCP2	3			MEI1	2	TPX2	4	KIF1C	2
		TFRC	3			MEIS1	2	TYK2	4	LY6D	2
		TK1	3			NR1D2	2	TYMS	5	MAOA	2
		TPX2	3			PIK3R3	2	UBE2C	3	PITX2	2
		TYK2	3			PSIP1	2	USP1	4	PKP3	2
		TYMS	3			SLFN13	2			S100P	2
		UBE2C	3			SMARCA2	2			SERPINB4	2
						SYCP2	4			SLC24A3	2
						SYNGR3	3			UGT1A6	2
						TAF7L	2			WNT4	2
						TCAM1	2				
						TYMS	2				
						USP1	2				
						WDR76	2				
						ZNF238	2				

**Table 3:** Common Deregulation between HPV-Infected Mucosal Tumors and HPV Models

Model	Study	HPV-Signature	Common Genes
CC progression	Gius 2007	Signature 5	CDKN2A, CENPF, MCM2, RFC4 and TK1
		Signature 6	CDA, CRABP2, KIF1C and KLK11
Human Keratinocytes	Duffy 2003	Signature 5	None
		Signature 6	KLK7, ECM1, LY6D and SULT2B1
	Wan 2008	Signature 5	CDKN2A
		Signature 6	KLK7, KLK10, KLK11, LY6D, SCEL and S100P
Organotypic skin	Garner-Hamrick 2004	Signature 5	CDC7, CDKN2A, DHFR, EZH2, MCM2, MELK, PCNA, RFC4, TPX2, TYMS and USP1
		Signature 6	CDA, EIF5A, KLK8 and KLK13
Hela cell line	Kuner 2007	Signature 5	CENPF, DTL, EZH2, KIF15, MCM2, MELK and PSMB9
		Signature 6	HIST2H3PS2
Transgenic mice	pRb/p107/p130	Signature 5	DHFR, E2F1, LIG1, MCM2, PCNA, RFC4, TYMS and USP1
		Signature 6	NA
	pRb/p53	Signature 5	CDC7, ECT2, MCM2, TK1, TPX2, TYMS and UBE2C
		Signature 6	NA

NA: non applicable.

## ABBREVIATIONS

HPV: = Human papillomavirus
pRb: = Retinoblastoma protein
CC: = Cervical carcinoma
HNSCC: = Head and neck squamous cell carcinoma
LSIL: = Low-grade squamous intraepithelial lesion
HSIL: = High-grade squamous intraepithelial lesion
CIN: = Carcinoma in situ
GO: = Gene Ontology
